# Evaluation of reliability and validity of the Serbian Aphasia Screening Test

**DOI:** 10.1371/journal.pone.0304565

**Published:** 2024-05-31

**Authors:** Mile Vuković, Tanja Milovanović, Predrag Teovanović, Vesna Stojanovik

**Affiliations:** 1 University of Belgrade, Faculty of Special Education and Rehabilitation, Belgrade, Serbia; 2 Rehabilitation Clinic “Dr Miroslav Zotović”, Belgrade, Serbia; 3 School of Psychology and Clinical Language Sciences, University of Reading, Reading, United Kingdom; Basque Center on Cognition Brain and Language, SPAIN

## Abstract

**Purpose:**

A rise in strokes worldwide means that the number of people affected by aphasia is increasing. Early and accurate diagnosis of aphasia is crucial for recovery. Presently, there are no dedicated screening tests tailored for evaluating aphasia in Serbian-speaking individuals. This paper presents and describes the psychometric properties of the Serbian Aphasia Screening Test (SAST), a novel aphasia screening tool designed specifically for Serbian speakers. This initiative fills the gap in aphasia assessment tools for the Serbian population, providing a comprehensive and culturally sensitive approach to the evaluation of language disorders.

**Method:**

Data using the SAST were collected from 240 participants: 120 Serbian speakers with aphasia after stroke compared to 120 neurotypical individuals. The assessment included the following subtests: conversation, verbal automatized sequences, auditory comprehension, visual confrontation naming, responsive naming, repetition of words, repetition of sentences, oral word reading, oral sentence reading, reading comprehension, and writing. The main objectives were to ascertain the psychometric qualities of the SAST, including inter-rater reliability of scoring, test-retest reliability, reliability of the individual subtests, overall test reliability, and inter-correlations among subtests. Additionally, the study evaluated the discriminatory capability of the SAST in distinguishing between individuals with aphasia and neurotypical controls, as well as between individuals with different types of aphasia.

**Results:**

The findings revealed that the SAST has excellent inter-rater reliability, test-retest reliability, and internal consistency. There were statistically significant differences between individuals with aphasia and neurotypical controls on all SAST subtests. Furthermore, the study identified significant differences in language profiles among participants with different types of aphasia. The significant correlations between scores on the SAST and on the Boston Diagnostic Aphasia Examination (BDAE) suggest good convergent validity of the SAST.

**Conclusions:**

The results underscore the robust psychometric properties of this novel screening assessment (SAST) and its ability to effectively discriminate between diverse linguistic abilities within different aphasia syndromes in Serbian speaking individuals.

## 1. Introduction

There has been a rise in stroke worldwide which means that there are more people acquire aphasia [1], with the condition affecting approximately 20% to 40% of stroke survivors [2]. A number of different tools are used for assessing language and communication in acquired aphasia, including screening tests, comprehensive aphasia tests, bedside clinical examinations, and tests focusing on specific linguistic functions [3]. Screening tests aim to quickly and efficiently detect the presence or absence of aphasic disorder without delving into detailed description of the disorder or providing a description of any single language skill. Clinicians typically employ these tests during a first meeting with the patient in the acute stage, or at any other stages of recovery when a more comprehensive examination might be too demanding. Prominent screening tests include the *Frenchay Aphasia Screening Test* (FAST) [4, 5], *ScreeLing Test* [6], *Acute Aphasia Screening Protocol* [7], *Mississippi Aphasia Screening Test* [8] and *Reitan-Indiana Aphasia Screening Test* [9]. These tests vary in administration difficulty; for example, the FAST can be administered by a clinician without training as a speech and language therapist (SLT) in about 10 minutes for individuals with acute or chronic aphasia. In contrast, the ScreeLing test requires expertise and training and can only be used by qualified SLTs [10]. The administration time is 15 minutes and it provides information on an individual’s linguistic abilities in order to guide early treatment decisions.

Comprehensive aphasia tests seek to obtain a diverse range of performance at different levels of task difficulty and encompass all language dimensions relevant to language disability. These assessments commonly evaluate naming, spontaneous speech, oral expression, auditory comprehension, repetition, reading, and writing through an organized set of tasks [11]. These tests are administered usually after the acute stage, provided that the patients are able to undergo the testing procedure.

Many comprehensive aphasia assessment batteries have been published, with two enduringly prominent options being the *Boston Diagnostic Aphasia Examination*, now in its third edition [12, 13] and the *Western Aphasia Battery-Revised* (WAB-R) [14, 15]. Both have in common the goals of classifying an individual into classical aphasia subtypes and assessing aphasia severity based on response patterns on several subtests. Recently, Wilson et al. [16] described a new assessment, the *Quick Aphasia Battery for English*, offering a balance between length and comprehensiveness. Furthermore, some comprehensive batteries, such as the BDAE, have a short form, which is still more comprehensive than a screening test [12].

Additionally, *Bedside Clinical Examination* (BCE) has traditionally been used as a clinical tool, offering a brief evaluation of language in hospitalized patients, primarily during the acute phase following a stroke. However, there are limitations to BCE. Firstly, its administration can vary between different examination settings, both in context and in the way in which it is administered. Secondly, the assessment heavily relies on the subjective judgment of the clinician. Thirdly, BCE results pose challenges for replication and comparison due to the absence of specific procedures and large variability with regard to its sensitivity and specificity [11, 17].

Finally, there are tests of specific language functions, such as the *Boston Naming Test*–BNT [18], or the *Token Test*–TT [19], focus on detailed measurement of particular aspects of language. These language functions may encompass object naming, auditory comprehension, reading comprehension, etc. For example, the BNT is a widely used test for visual confrontation naming, which employs stimuli of increasing difficulty, ranging from simple, high-frequency vocabulary to rare words. On the other hand, the TT is a brief test used to examine subtle auditory comprehension difficulties in aphasic patients. Generally speaking, tests of specific language functions offer several advantages, including a deeper evaluation of specific language behaviour or response modality, an examination of behaviour in individuals who are likely to score at ceiling or floor on a comprehensive aphasia battery, a presentation of a broader range of stimulus items in an area compared to what is typically included in a comprehensive aphasia battery [3].

Although screening tests are primarily used in the acute phase after stroke, they can also be used in the subacute phase and among patients with chronic aphasia, especially when exhaustive testing with large comprehensive test batteries proves to be impractical or impossible [20]. On the other hand, comprehensive test batteries are designed to assess in detail the different domains of language, often requiring multiple assessments of the same patient. Repeat testing can often be frustrating, especially for patients who have insight into their disorder and whose recovery of language skills is not in line with their expectations. Therefore, it is crucial to have a brief and easy screening test for aphasia that may be administered by speech and language therapists, and in exceptional cases by other health professionals, with the clinical aim of providing a quick screen of a person’s language abilities. Screening tests for aphasia may also be used for research purposes. In addition, advice regarding communication may be better personalized using results from screening tests [10].

Various validated screening tests are utilized worldwide, and a recent advancement in Serbia involves the creation of a clinical research tool specifically designed to assess word-reading abilities in individuals with acquired aphasia [21]. Although the BDAE, a comprehensive battery of tests, had been translated into Serbian for the assessment of aphasia, it remains a literal translation rather than an adaptation. Literal translations are never ideal because there is hardly ever a one-to-one match with regard to vocabulary and syntactic structures between any two languages [22]. Furthermore, translated items are often culturally not appropriate or may include unfamiliar concepts. For example, a sentence from the BDAE “*The phantom soared across the foggy heath”* was translated literally as “Fantom se vinuo visoko nad maglovitu pustaru” [23], which may not resonate as naturally in Serbian, given the uncommon geographical context and theme. The term ‘heath’ does not denote a natural scenario that is commonly known to Serbian speakers as heaths are not an integral part of the geographical landscape of Serbia and ghosts are not typically part of stories in Serbia. An another example from the BDAE, “The Chinese fan had a rare emerald” was translated as “Na kineskoj lepezi je bio redak smaragd”, which is, culturally speaking, an unusual picture not routinely associated with people’s daily life in Serbia.

Given the absence of standardised language measures to diagnose aphasia in Serbian, and the importance of people having a language assessment after a stroke to optimise recovery outcomes. the *Serbian Aphasia Screening Test* (SAST) was developed by Vuković [24] as a screening tool specifically designed for Serbian speakers (see S1 File). The sentences and phrases chosen in the SAST are culturally relevant, incorporating concepts and pictures familiar in the Serbian context. The SAST allows for a short and quick assessment of language modalities to identify the presence of aphasic disorders. By comparing language abilities in conversation, auditory comprehension, naming, repetition, reading, and writing, clinicians can gain insights into the presence, type, and severity of aphasia.

This study aims to provide a comprehensive description of the Serbian Aphasia Screening Test and present its psychometric properties as it applies to patients in the post-acute and chronic phase of aphasia who were at least one month post-onset. The paper also reports the screening assessment data of Serbian speakers with aphasia after stroke compared to neurotypical individuals.

The primary objectives of this study were fivefold. Firstly, we aimed to assess whether the SAST demonstrates acceptable psychometric quality when applied to people with aphasia. This includes evaluating inter-rater reliability in scoring, test-retest reliability, reliability of individual subtests, overall test reliability, and inter-correlations among individual subtests. Secondly, we investigated whether the SAST could discriminate between people with aphasia and neurotypical controls. Thirdly, we explored whether participants’ gender, age, education, and time post-onset affect SAST performance. Fourthly, we examined if SAST is sensitive to distinguish between people with different types of aphasia and lesion sites. Finaly, we compared performance on the SAST with performance on the BDAE.

## 2. Method

This study was conducted in accordance with the ethical standards specified in the Declaration of Helsinki, and the protocol was approved by the local human research ethics committee. The study was conducted at the Clinic for Rehabilitation Dr Miroslav Zotović, Belgrade, Serbia between 2017 and 2020. All participants voluntarily agreed to participate in the study and provided informed consent.

### 2.1. Participants

There were two distinct groups of participants in our study: a clinical group, represented by people with aphasia (PWA), also referred to as patients, and a control group (CG), comprising neurotypical individuals.

The PWA group consisted of 120 patients with stroke-induced aphasia. These participants were at least one month post onset (*M* = 13.45 months; *SD =* 16.73), positioned in the post-acute and chronic phases. The inclusion criteria for the clinical group were as follows: 1) the presence of aphasia caused by a single cerebrovascular stroke (CVI) in the left hemisphere, 2) at least one month after onset of CVI, 3) the participant is cooperative to the extent that allows testing, 4) right-handedness, 5) age > 18 years, 6) neurotypical premorbid speech and language abilities, and 7) Serbian as the mother tongue.

The CG consisted of 120 neurologically healthy participants with no history of mental health disorders, speech impairment, or language impairment. This group consisted of individuals employed at the rehabilitation clinic Dr Miroslav Zotović, Belgrade, Serbia, who volunteered to participate.

The general characteristics of the participants are presented in Table 1. Notably, PWA and CG did not differ significantly in terms of gender, age, or education level.

**Table 1:** General characteristics of the two groups of participants.

	PWA (n = 120)	CG (n = 120)	Test of difference
Age (years): M (SD)	61.38 (11.80)	61.43 (11.96)	*t*(237) = 0.03, *p* = .97
Minimum age	31	31	
Maximum age	84	83	
Years of education: M (SD)	12.35 (2.70)	12.39 (2.56)	*t*(237) = 0.12, *p* = .90
Min. years of education	4	4	
Max. years of education	19	18	
Gender			
Female n (%)	56 (46.7%)	52 (43.3%)	χ^2^(1) = 0.27, *p* = .60
Male n (%)	64 (53.3%)	68 (56.7%)	

*Note*. PWA—people with aphasia; CG—control group

#### 2.1.1. Distribution of patients according to aphasia type and severity

All participants were assessed using the translated version of the BDAE, as this is the most common assessment tool used with Serbian aphasic patients [13]. They were all also administered the SAST. The participants were subsequently categorized into specific aphasia subgroups based on the assessment outcomes.

The largest number of participants had Broca’s aphasia (30.8%), followed by global aphasia (15.8%), transcortical motor aphasia (TMA) (10.8%) and both Wernicke’s and subcortical motor aphasia (SMA) at 8.3% each. The smallest number of participants had anomic, conduction or transcortical sensory aphasia (TSA) (5.8% each). For 8.3% of the participants, language function disorders did not correspond to any type of classical aphasic syndrome, which is why they were classified under nonspecific aphasia (unclassifiable group).

In addition, the severity of aphasia was evaluated according to the BDAE Aphasia Severity Rating scale, and patients were categorized into four groups: 1) very severe aphasia, where all communication is through fragmentary expression and a great deal of inference, questioning, and guessing is required by the listener, 2) severe aphasia, where conversation about familiar topics is possible with the help of the listener, 3) moderate aphasia, where the patient can discuss everyday topics with little or no help, but difficulties with speech and/or comprehension makes a conversation about certain topics difficult or impossible, and 4) mild aphasia characterised by some obvious loss of fluency of speech or comprehension, without noticeable limitations on ideas expressed or form of expression. The largest proportion of participants had a severe and moderate form of aphasia (70%), followed by mild aphasia, and then by very severe aphasia. Table 2 shows the distribution of participants into different aphasic subgroups and different levels of severity of aphasia.

**Table 2:** Distribution of participants across different aphasic subgroups and severity levels of aphasia, based on the total raw BDAE scores and on the Aphasia Severity Rating Scale.

Type of Aphasia	Raw scores on the BDAE (Maximum Score that can be obtained: 545)				Aphasia Severity Rating Scale			
	Min	Max	M	SD	Very severe aphasia	Severe aphasia	Moderate aphasia	Mild aphasia
Broca’s (n = 37)	98	400	234.49	77.65	10.9%	35.1%	35.1%	18.9%
Anomic (n = 7)	364	481	441.86	38.68	0%	0%	0%	100%
Transcortical sensory (n = 7)	127	258	180.71	56.96	14.3%	71.4%	14.3%	0%
Transcortical motor (n = 13)	168	441	325.31	82.03	0%	30.8%	30.8%	38.4%
Conduction (n = 7)	177	413	271.57	88.32	14.3%	42.8%	14.3%	28.6%
Subcortical motor/anterior (n = 10)	310	447	382.60	40.47	0%	0%	10%	90%
Wernicke’s (n = 10)	153	210	170.10	15.83	50%	30%	20%	0%
Nonspecific / Unclassified (n = 10)	26	64	42.10	13.54	90%	10%	0%	0%
Global (n = 19)	NA	NA	NA	NA	100%	0%	0%	0%

*Note*. NA—In participants with global aphasia, it was not possible to administer the BDAE.

#### 2.1.2. Distribution of participants according to the site of the lesion

The location of the lesion was determined based on computed tomography (CT) or magnetic resonance imaging (MRI) of the brain. According to these data, the patients in this study were classified into four categories: 1) anterior cortical lesion, 2) posterior cortical lesion, 3) anterior-posterior cortical lesion, and 4) subcortical lesion. The group with anterior lesions included participants with lesions in the frontal areas of the cortex, and the group with posterior lesions included participants with lesions in the temporal, temporo-parietal, parieto-occipital, temporo-occipital, and parieto-temporo-occipital areas. The group with anterior-posterior lesions consisted of participants with frontal-temporal, frontal-parietal, and frontal-temporo-parietal lesions. The fourth group included patients with anterior lesions in subcortical areas (anterior/putaminal lesions). More detailed data on the distribution of participants according to site lesion are presented in in Table 3.

**Table 3:** Distribution of participants across site of lesion and severity of aphasia (row percentages).

Site of lesion (total = 120)	Severity of Aphasia			
	Very severe (n = 40)	Severe (n = 28)	Moderate (n = 22)	Mild (n = 30)
Anterior cortical (n = 20; 16.7%)	10.0%	20.0%	40.0%	30.0%
Posterior cortical (n = 27; 22.5%)	29.6%	33.3%	11.1%	26.0%
Anterior-posterior cortical (n = 66; 55.0%)	45.4%	21.2%	16.7%	16.7%
Subcortical lesion (n = 7; 5.8%)	0.0%	14.3%	0.0%	85.7%

### 2.2. Instruments and procedure

Serbian is a highly inflected, pro-drop language, with rich inflectional and derivational morphology. For this reason, we included different types of nouns when designing our test: feminine, masculine and neuter and also, we ensured that nouns and adjectives had different case morphology (nominative, accusative, dative, locative, instrumental). Also, when choosing test tasks, we were guided by the grammatical features of the Serbian language: case morphology on nouns and adjectives; use of reflexive verbs; active adjectival participles (used to form past tense). In addition, the frequency of the words chosen was taken into consideration such that nouns were chosen to represent different frequencies. As this is a screening test with a limited number of items, the nouns were not counterbalanced for gender or case, but we there was a representation of the different genders and most cases). The result was the *Serbian Aphasia Screening Test*–SAST [24] which allows for a short and quick assessment of language skills in order to determine the presence of aphasic disorders. SAST includes a subtest for conversation and 10 additional subtests. The SAST assessment began with a conversation with the patient by asking them questions about themselves—name and surname, place of residence, occupation, and reason for visiting the clinic or rehabilitation center. If the participants did not answer the question immediately, the examiner helped them with additional questions. For example, if the patients did not answer the question which asked where they live, the examiner would ask a second question giving two different towns as alternatives, one of which was the participant’s place of residence. The examiner aimed to gain a clinical impression of language ability through a short conversation. The answers provided by the patients during the conversation were not scored. Nevertheless, the test allows the examiner to note down difficulties in auditory comprehension, non-fluent speech, the presence of paraphasia, total number of words etc.

After the conversation, the examiner administered the test tasks, and these were scored according to the test instructions. In addition to the scores achieved on the subtests and the total score on the SAST, the total number of words obtained from the conversation subtest was also analysed. The SAST took between 10 to 20 minutes to administer. Below is a description of the 10 SAST subtests which were scored.

1. *Automatized Sequences*. The participants were required to count from one to twenty-one and then list the days of the week. If a participant was unable to perform these tasks spontaneously, the examiner helped them by saying what the first number was or what the first day of the week was. Each completed task (spontaneously or with support) was given one point. The patient could achieve a maximum of two points on this subtest.
2. *Auditory Comprehension*. Participants were asked to perform five verbal commands. These varied in length and complexity, such that the first three commands ask the person to understand one information unit and the structure is Verb (V) Object (O) where the O is a single word noun. The fourth command contained two information units and the structure was Adjunct (A), V, O and the noun phrases for A and O contained two elements (adjective + noun). The fifth item contained three information units and the structure was V, O (where the O is a single noun) for the 1^st^ information unit, V O (where the O is a single noun) for the second information unit and A V for the third one. Item repetition was not permitted for the first three commands. One point was given for each successful response. The patient was then asked to perform the remaining two commands. The examiner would repeat the fourth and fifth verbal commands if the patient required repetition. If the participants performed the commands successfully, they received two points for the fourth command and three points for the fifth command. The maximum score on this subtest was eight.
3. *Visual Confrontation Naming*. The participants were shown 10 pictures (colour photographs), ordered according to frequency (from more frequent, like a watch, to the lower frequency ones, such as a lighter or a paperclip) and they were asked to name each picture. Words for naming were selected to reflect a range of frequency in written Serbian [25]. Each successfully named picture was given one point. The maximum score for this task was 10. See the Appendix for details of the specific words used.
4. *Responsive Naming*. The examiner asked stimulus questions (e.g. “What do we cut bread with?”) that required one-word responses (e.g. “Knife*”*). One point was awarded for each completed item. The maximum score on this subtest was two.
5. *Word Repetition*. The participants were asked to repeat one word at a time after the examiner. Each successfully repeated word was given one point. The maximum possible score for this task was 10. See the Appendix for details about the words used.
6. *Sentence Repetition*. The participants were asked to repeat one sentence at a time after the examiner. The first sentence was simple (Adjunct, Verb, Complement) whereas the second sentence was complex, containing coordination of two clauses. If the participant was unable to repeat the sentence after the first attempt, the examiner could repeat it, only if the participant requested it. Each completed task was given one point. The maximum score on this subtest was two.
7. *Word reading*. The participants were shown a card that contained a word and asked to read it. Each successfully read word was given one point. The maximum score on this subtest was 10. See the Appendix for details about the cards used.
8. *Oral sentence reading*. The participants were shown a sentence printed on a card and were asked to read it. Each correctly read sentence was given one point. The maximum score on this subtest was two.
9. *Reading comprehension*. The participants were shown written commands and were asked to read one command at a time and perform the action (e.g. “raise your arm”). Each completed task was given one point. The maximum score on this subtest was two points.
10. *Writing*. The participants were given a blank sheet of paper and asked to write their names first and then to write a sentence of their choosing. Each completed task was given one point. The maximum score on this subtest was two points. Points were not deducted if a participant’s handwriting was impaired due to their use of a non-dominant hand, or due to a motor deficit of the hand.

A speech and language therapist administered the screening test to all participants. The testing was done in a quiet room, at a time of day when the patient was most ready for testing. The same procedure was used for both participant groups. The assessment was performed at the clinic or in the participants’ homes.

After the SAST, the translated version of the Boston Diagnostic Aphasia Examination [13] was administered to all participants by the same speech and language therapist who performed the SAST. Based on the SAST and the BDAE results, and following the consensus between two speech-language therapists (the first two authors of this study), the participants were classified into specific categories of aphasic syndromes (Table 2). Patients whose clinical picture of aphasia did not correspond to any known aphasic syndrome were classified as having nonspecific (unclassified) aphasia (Table 2).

Furthermore, based on the clinical assessment of spontaneous speech (conversation), the patients were divided into two groups: fluent and non-fluent aphasia. The patients with Broca’s, transcortical motor, subcortical motor, global and nonspecific aphasias were classified as non-fluent, while patients with anomic, conduction, transcortical sensory, and Wernicke’s aphasia were classified as fluent.

#### 2.2.1 Evaluation of reliability of SAST

To ensure inter-rater reliability, a second scorer blinded to the first rater’s scoring rated all responses for the presence or absence of errors. If an error was present, they indicated the type of the error. To ensure intra-rater reliability, the first scorer re-rated all the responses that were blind to the original scoring. Intraclass correlations for inter- and intra-rater agreement on the presence or absence of errors and a class of errors were all highly significant (*r* = .99; *p <* .001).

To measure test-retest reliability, a group of 20 chronic patients (11 female; 9 male) with aphasia (at least six months post-onset) were assessed six weeks after the first assessment. They were aged between 41 and 78 years (*M* = 60.00 years, SD = 10.40). Participants with all types of aphasia and all levels of aphasia severity were included in this group.

### 2.3. Data analysis

In order to address the first objective which was whether the SAST has acceptable psychometric qualities, we examined the Cronbach’s measures of internal consistency of score and computed correlation coefficients to analyse test–retest reliability for SAST scores; we used correlation coefficients and principal component analysis (PCA) to test the convergence of SAST indicators. To address the second objective (i.e. whether the SAST can distinguish between people with aphasia and neurotypical controls), we ran t-tests which compared the SAST scores of the PWA group to those of the control group. Given that all control group participants were at ceiling, we carried out one-sample t-tests and determined measures of effect size (Cohen’s d) as the difference between the maximum and observed achievement in relation to the observed variability of the PWA group. To address the third objective (effect of gender, age, education and time post-onset on SAST scores) we ran a regression analysis with gender, age, education and time post-onset as predictors of SAST subscales scores. To address the fourth objective of the study (whether the SAST was sensitive to distinguish between people with different types of aphasia and lesion site), one-way ANOVA was run. In order to find out how participants with different levels of severity of aphasia perform on different subtests of the SAST, we also used one-way ANOVA. Finally, to address the fifth objective, Pearson’s corelation coefficients were run to examine the relationship between SAST and BDAE scores.

The data analyses were carried out with the Statistical Package for Social Sciences–SPSS for Windows, version 23.0. Specific details of the statistical tests used are provided in the Results section.

## 3. Results

### 3.1. SAST psychometric qualities—test reliability and convergence of indicators

Table 4 shows the descriptors of scores distributions and reliability measures on the SAST subtests and the screening test as a whole for subset of PWA participants. The analysis revealed satisfactory to high levels of reliability for all subtests ranging from .76 to .98, except for the sentence repetition subtest (α = .61). There was a high level of reliability for the SAST total score (α = .98). Similarly, test-retest reliabilities for 20 participants diagnosed with chronic aphasia who were tested again after six weeks were well above .80, except for sentence repetition (r = .74), with some being the same at both time points (e.g. auditory comprehension, oral sentence reading, and writing).

**Table 4:** Description and reliability of the SAST subtests, and the test as a whole on subset of PWA (n = 120).

SAST subtests	Range	M	SD	Sk	Ku	No. of items	Cronbach’s α	Test-retest r(18)
Automatized sequences	0–2	1.28	0.84	-0.56	-1.36	2	.78	.87
Auditory comprehension	0–8	5.43	2.60	-0.79	-0.55	5	.76	1
Visual confrontation naming	0–10	3.84	3.80	0.43	-1.38	10	.94	.99
Responsive naming	0–2	0.92	0.91	0.15	-1.79	2	.81	.95
Word repetition	0–10	5.78	4.12	-0.42	-1.55	10	.97	.98
Sentence repetition	0–2	0.72	0.77	0.51	-1.12	2	.61	.74
Word reading	0–10	4.49	4.29	0.15	-1.76	10	.97	.98
Oral sentence reading	0–2	0.68	0.68	0.69	-1.44	2	.93	1
Reading comprehension	0–2	0.90	0.90	0.20	-1.83	2	.88	.99
Writing	0–2	0.47	0.47	1.23	-0.05	2	.79	1
**Total score**	0–50	24.66	16.82	0.02	-1.46	47	.98	1

Details of the inter-correlations of the SAST subscales/subtests are presented in Table 5. These results indicate that scores on subscales were highly correlated and significant (all *p*s < .001).

**Table 5:** Intercorrelations among SAST subtests and overall score.

Subtests	1	2	3	4	5	6	7	8	9	10
1. Automatized sequences										
2. Auditory comprehension	.67									
3. Visual confrontation naming	.69	.78								
4. Responsive naming	.71	.73	.86							
5. Word repetition	.75	.71	.80	.80						
6. Sentence repetition	.69	.67	.85	.80	.84					
7. Word reading	.70	.73	.89	.78	.82	.82				
8. Oral sentence reading	.62	.69	.85	.78	.74	.79	.91			
9. Reading comprehension	.66	.77	.81	.71	.70	.73	.87	.85		
10. Writing	.60	.69	.83	.73	.69	.77	.76	.79	.78	
11. Total score	.77	.84	.96	.89	.90	.89	.95	.89	.87	.83

Furthermore, the first principal component extracted from the 10 subtest scores had an eigenvalue 7.86, i.e. it explained 78.6% of the variance of the measures. Scores from all subtests very highly loaded by the first principal component (all above .75; see Table 6). Such pattern of results suggests a high degree of convergence among various aspects of communication and language skills assessed by the SAST, indicating that they measure the same underlying ability.

**Table 6:** Saturations on the first main component.

SAST subtests	Loadings
Automatized sequences	.79
Auditory comprehension	.84
Visual confrontation naming	.95
Responsive naming	.89
Repetition of words	.88
Sentence repetition	.90
Word reading	.94
Oral sentence reading	.91
Reading comprehension	.89
Writing	.86

### 3.2. Comparison between PWA and CG on SAST

The following analyses compared the performance on the SAST between PWA and CG. The results presented in Table 7 show that the PWA scored significantly lower on all subtests and had a significantly lower overall score than those in the control group. Overall differences between two group were around 1.50 standard deviation but it ranged between 0.79 (for automatized sequences) and 6.07 (for visual naming) across subtests.

**Table 7:** Differences between participants with aphasia (PWA) and control participants (CG) on the subtests and overall SAST score.

SAST	PWA (N = 120)		CG (N = 120)		Difference Test		Effect size
**Subtests**	M	SD	Mean	SD	t (119)	p	d
Automatized sequences	1.28	0.84	2	-	8.65	< .001	0.79
Auditory comprehension	5.43	2.60	8	-	10.24	< .001	0.93
Visual naming	3.84	3.80	10	-	17.39	< .001	6.07
Responsive naming	0.92	0.91	2	-	12.73	< .001	1.05
Repetition of words	5.78	4.12	10	-	10.89	< .001	4.11
Sentence repetition	0.72	0.77	2	-	18.02	< .001	1.28
Word reading	4.49	4.29	10	-	13.84	< .001	5.50
Oral sentence reading	0.68	0.68	2	-	16.93	< .001	1.37
Reading comprehension	0.90	0.90	2	-	13.52	< .001	1.23
Writing	0.47	0.47	2	-	22.37	< .001	2.04
**Total score**	24.66	16.82	50	-	16.50	< .001	1.51

### 3.3. Effect of gender, age, education and time post-onset on SAST performance

We examined the relationship between the SAST scores and gender, age, education and time post-onset using a regression analysis. This is presented in Table 8.

**Table 8:** Results of regression analyses (Total score and subtest scores of the SAST).

SAST subtests	F(4, 115)	Adjusted R^2^	β_Gender_	β_Age_	β_Education_	β_TPO_
Automatized sequences	1.60	2.0%	.10	-.07	-.11	.16†
Auditory comprehension	1.87	2.9%	.06	-.08	.01	.22*
Visual naming	1.72	2.4%	-.01	-.04	.08	.22*
Responsive naming	3.92**	8.9%	-.07	-.02	.16†	.30***
Word repetition	0.71	0.0%	.01	-.03	.05	.14
Sentence repetition	0.53	0.0%	.00	.00	.06	.12
Word reading	1.98	3.2%	-.04	.08	.07	.24*
Oral sentence reading	2.38†	4.4%	-.02	-.05	.09	.25**
Reading comprehension	0.94	0.0%	-.08	-.03	.03	.15
Writing	1.26	0.1%	-.01	-.14	.08	.12
**Total Score**	1.96	3.1%	-.02	-.02	.07	.24**

*Note*. TPO–Time post-onset

†p < .10

*p < .05

**p< .01

***p< .001

The results of the regression analysis reveal that a set of predictor variables, including gender, age, education, and time post onset (TPO), did not significantly account for substantial portion of the variance in both total and subtest scores. An exception to this trend was observed in the responsive naming task, where these predictors accounted for 8.9% percent of variance. For all other cases, the predictor set explained less than 5% of the criterion variance (*F*s < 2.50, *p*s > .05).

However, it should be noted that time since injury in months emerged as a sporadically significant partial predictor of SAST performance, particularly in the responsive naming task, oral sentence reading, word reading and the total score. Conversely, none of the remaining predictors contributed significantly to the regression model in any analysis. This pattern of results indicates that SAST performance remains relatively invariant concerning gender, age, and education. However, it is sensitive to the time post onset, with scores improving over time since the injury, which is in accordance with our initial expectations.

### 3.4. Performance on the SAST in relation to different levels of severity and types of aphasia

Table 9 presents the averages and standard deviations of performance on the SAST subtests and the total scores of PWA with different levels of aphasia severity.

**Table 9:** Differences on the subtests of the SAST and the total score between participants with different levels of severity of aphasia.

SAST subtests			Automatized sequences	Auditory comprehension	Visual naming	Responsive naming	Repetition of words	Sentence repetition	Word reading	Oral sentence reading	Reading comprehension	Writing	Total score
**Group**			**Mean (SD) for rows 1–4**										
**Severity of aphasia**	**1**	Mild (n = 30)	1.83 (0.37)	7.60 (0.71)	8.97 (1.15)	1.80 (0.48)	9.70 (0.59)	1.57 (0.56)	9.60 (0.85)	1.87 (0.43)	1.93 (0.25)	1.23 (0.86)	46.17 (2.92)
	**2**	Moderate (n = 22)	1.68 (0.47)	6.64 (1.32)	5.50 (2.70)	1.41 (0.73)	8.09 (2.50)	0.95 (0.65)	7.41 (2.46)	0.91 (0.86)	1.18 (0.90)	0.57 (0.60)	35.50 (3.08)
	**3**	Severe (n = 29)	1.59 (0.68)	5.59 (1.57)	2.48 (2.13)	0.86 (0.83)	6.86 (3.02)	0.63 (0.55)	3.30 (2.97)	0.14 (0.35)	0.69 (0.59)	0.29 (0.21)	20.76 (4.86)
	**4**	Very severe (n = 39)	0.63 (0.38)	2.97 (2.66)	0.58 (0.23)	0.35 (0.08)	1.84 (0.97)	0.22 (0.05)	0.22 (0.05)	0.03 (0.16)	0.10 (0.08)	0.10 (0.05)	4.92 (3.45)
F(3, 116)			67.80	40.33	170.99	52.62	100.18	40.74	171.07	89.31	53.91	25.77	780.13
p			< .001	< .001	< .001	< .001	< .001	< .001	< .001	< .001	< .001	< .001	< .001
η^2^			.63	.52	.82	.58	.72	.54	.82	.70	.58	.40	.95
Statistically significant Scheffe’s post hoc differences between groups with different severity			Groups 1–4; 2–4; 3–4	Groups 1–3; 1–4; 2–4; 3–4	Groups 1–2; 1–3; 1–4; 2–3; 2–4; 3–4	Groups 1–3; 1–4; 2–3; 2–4; 3–4;	Groups 1–3; 1–4; 2–4; 3–4	Groups 1–2; 1–3; 1–4; 2–4; 3–4	Groups 1–2; 1–3; 1–4; 2–3; 2–4; 3–4	Groups 1–2; 1–3; 1–4; 2–3; 2–4	Groups 1–2; 1–3; 1–4; 2–3; 2–4; 3–4	Groups 1–2; 1–3; 1–4; 2–4	Groups 1–2; 1–3; 1–4; 2–3; 2–4; 3–4

Note: SD–standard deviation

The results in Table 9 show that the mean values of the SAST subtests and the overall scores decreased with increasing aphasia severity. Participants with mild aphasia had the highest average scores, whereas those with very severe aphasia had the lowest average scores. The participants with mild aphasia differed from participants with very severe aphasia on all subtests by scoring significantly higher; however, there was a variable picture with regard to differences between those with mild and those with moderate aphasia, or those with moderate and those with severe aphasia. Full details are provided in Table 9 above.

Significant effect of the severity of aphasia was detected on the ***total SAST scores*** F(3, 116) = 780.13, *p* < .001). ***Post-hoc analysis*** revealed that the patients with mild aphasia were significantly better than those with severe, very severe and moderate aphasias (*p* < .001, each). The patients with moderate aphasia were significantly better than those with severe and very severe aphasias (*p* < .001). The patients with severe aphasia were significantly better than those with very severe aphasia (*p* < .001).

In addition to the differences in the total score, significant differences between aphasia subgroups on ***each subtest of the SAST*** were also found (Table 10).

**Table 10:** Differences on the subtests of the SAST and the total score between participants with different types of aphasia.

SAST subtests	Automatized sequences	Auditory comprehension	Visual naming	Responsive naming	Repetition of words	Sentence repetition	Word reading	Sentence reading	Reading compre hension	Writing	Total score
**Type of aphasia**	**Mean (S.D.) for rows 1–9**										
1. Broca’s (n = 37)	1.59 (0.64)	6.41 (1.26)	3.32 (2.58)	1.30 (0.81)	6.89 (3.32)	0.65 (0.48)	5.24 (3.88)	0.84 (0.88)	1.08 (0.83)	0.43 (0.68)	29.03 (11.81)
2. Anomic (n = 7)	2.00 (0.00)	7.86 (0.38)	7.14 (1.03)	1.71 (0.48)	9.86 (0.38)	1.86 (0.37)	9.86 (0.38)	2.00 (0.00)	1.86 (0.38)	1.86 (0.38)	47.71 (2.43)
3. TSA (n = 7)	1.29 (0.95)	3.29 (1.49)	2.29 (3.4)	0.43 (0.78)	8.86 (1.46)	1.43 (0.53)	5.29 (4.11)	0.00 (0.00)	0.00 (0.00)	0.00 (0.00)	22.29 (7.85)
4. TMA (n = 13)	1.62 (0.65)	7.08 (1.32)	5.54 (3.15)	1.46 (0.77)	9.54 (0.88)	1.69 (0.48)	6.31 (3.48)	0.77 (0.93)	1.38 (0.87)	0.77 (0.83)	35.38 (10.63)
5. Conduction (n = 7)	1.57 (0.79)	7.43 (0.98)	4.43 (3.95)	1.14 (0.90)	6.29 (3.77)	0.29 (0.49)	5.14 (4.45)	0.71 (0.95)	1.57 (0.79)	0.57 (0.79)	29.14 (14.26)
6. SMA (n = 10)	1.80 (0.42)	7.70 (0.68)	9.20 (1.32)	1.90 (0.31)	9.70 (0.67)	1.50 (0.52)	9.40 (1.35)	1.80 (0.63)	2.00 (0.00)	1.00 (0.94)	46.50 (3.81)
7. Wernicke’s (n = 10)	0.90 (0.74)	3.90 (2.89)	1.50 (1.90)	0.10 (0.31)	2.10 (3.48)	0.20 (0.42)	2.50 (4.01)	0.30 (0.67)	0.60 (0.97)	0.10 (0.32)	12.80 (12.28)
8. Global (n = 19)	0.37 (0.67)	1.58 (1.46)	0.00 (0.00)	0.00 (0.00)	0.63 (1.30)	0.00 (0.00)	0.11 (0.31)	0.00 (0.00)	0.00 (0.00)	0.00 (0.00)	2.63 (1.98)
9. Nonspecific / Unclassified (n = 10)	0.50 (0.71)	4.70 (2.41)	0.70 (0.95)	0.30 (0.67)	2.20 (2.49)	0.10 (0.31)	0.00 (0.00)	0.00 (0.00)	0.00 (0.00)	0.20 (0.42)	8.80 (1.40)
F(8, 111)	10.10	27.68	18.26	15.31	25.04	26.17	14.54	11.17	16.59	8.71	34.53
p	< .001	< .001	< .001	< .001	< .001	< .001	< .001	< .001	< .001	< .001	< .001
η^2^	.42	.67	.57	.72	.65	.73	.50	.45	.54	.38	.71
Significant Scheffe’s post hoc test between different types of aphasia	1–8, 1–9; 2–8, 2–9; 3–6, 3–8, 3–9; 4–7, 4–8, 4–9; 5–7, 5-8-5-9; 6–7, 6–8, 6–9	1–3, 1–7, 1–8, 1–9; 2–3, 2–7, 2–8, 2–9, 3–4, 3–5, 3–6; 3–8, 3–9; 4–7, 4–8, 4–9; 5–7, 5–8, 5–9; 6–7, 6–8, 6–9; 7–9, 8–9.	1–2, 1–5, 1–7, 1–8, 1–9; 2–3, 2–7, 2–8, 2–9, 3–4, 3–6, 3–8, 3–9; 4–7, 4–8, 4–9; 5–6, 5–7, 5–8, 5–9, 6–7, 6–8, 6–9, 7–8	1–3, 1–7, 1–8; 1–9; 2–7, 2–8, 2–9; 3–4, 3–6; 4–7; 4–8; 4–9; 5–7, 5–8, 5–9; 6–7; 6–8; 6–9.	1–2, 1–3, 1–6, 1–7, 1–8, 1–9; 2–5, 2–7, 2–8, 2–9; 3–7, 3–8, 3–9; 4–5, 4–7, 4–8, 4–9; 5–7, 5–8, 5–9; 6–7, 6–8, 6–9.	1–2, 1–3, 1–4, 1–6, 1–8; 2–5; 2–7; 2–8, 2–9; 3–5, 3–6; 3–7, 3–8, 3–9; 4–5, 4–7, 4–8, 4–9; 5–6; 6–7, 6–8, 6–9.	1–2, 1–6, 1–7,1–8, 1–9; 2–7, 2–8, 2–9, 3–7,3–8,3–9; 4–7, 4–8, 4–9, 5–8, 5–9; 6–7, 6–8; 6–9.	1–2, 1–6, 1–8. 2–3; 2–7, 2–8, 2–9; 3–6; 4–6; 5–6, 6–7; 6–8, 6–9.	1–3, 1–6, 1–8, 1–9; 2–3, 2–8, 2–9, 3–4, 3–5, 3–6, 4–7, 4–8, 4–9, 5–8, 5–9, 6–7, 6–8, 6–9.	1–2, 2–3, 2–7, 2–8, 2–9; 6–8, 6–9	1–2, 1–3, 1–6, 1–7, 1–8, 1–9, 2–3,2–4, 2–5, 2–7, 2–8, 2–9, 3–4, 3–5, 3–6, 3–7, 3–8, 3–9, 4–6, 4–7, 4–8, 4–9, 5–6, 5–7, 5–8, 5–9, 6–7, 6–8, 6–9, 7–8.

Note: TSA–Transcortical sensory aphasia, TMA–Transcortical motor aphasia, SMA–Subcortical motor aphasia

On the ***Automatized sequences subtest***), post hoc analysis revealed that the patients with mild or moderate aphasia were significantly better than those with very severe aphasia (*p* < .001). The patients with severe aphasia were significantly better than those with very severe aphasia (*p* < .001).

On the ***Auditory comprehension subtest*** post hoc analysis revealed that the patients with mild aphasia were significantly better than those with severe aphasia (*p* < .001) and the patients with severe aphasia (*p* = .001). The patients with moderate or severe aphasia were significantly better than those with very severe aphasia (*p* < .001).

On the ***Visual naming subtest***, post hoc analysis revealed that the patients with mild aphasia were significantly better than those with moderate, severe and very severe aphasias (*p* < .001, each). The patients with moderate aphasia were significantly better than those with severe and very severe aphasia (*p* < .001). The patients with severe aphasia were significantly better than those with very severe aphasia (*p* < .001).

On the ***Responsive naming subtest*** post hoc analysis revealed that the patients with mild aphasia were significantly better than those with severe and very severe aphasias (*p* < .001, each). The patients with moderate aphasia were significantly better than those with severe aphasia (*p* = .006) and very severe aphasia (*p* < .001). The patients with severe aphasia were significantly better than those with very severe aphasia (*p* < .001).

On the ***Repetition of words subtest*** post hoc analysis revealed that the patients with mild aphasia were significantly better than those with severe and very severe aphasias (*p* < .001, each). The patients with moderate or severe aphasia were significantly better than those with very severe aphasia (*p* < .001).

On the ***Sentence repetition subtest***, post hoc analysis revealed that the patients with mild aphasia were significantly better than those with moderate aphasia (*p* = .007), severe and very severe aphasias (*p* < .001, each). The patients with moderate aphasia were significantly better than those with very severe aphasia (*p* < .001). The patients with severe aphasia were significantly better than those with very severe aphasia (*p* = .001).

On the ***Word reading subtest***, post hoc analysis revealed that the patients with mild aphasia were significantly better than those with moderate aphasia (*p* = .001), severe and very severe aphasias (*p* < .001, each). The patients with moderate aphasia were significantly better than those with severe and very severe aphasias (*p* < .001). The patients with severe aphasia were significantly better than those with very severe aphasia (*p* = .001).

On the ***Sentence reading subtest***, post hoc analysis revealed that the patients mild aphasia were significantly better than those with moderate, severe and very severe aphasias (*p* < .001, each). The patients with moderate aphasia were significantly better than those with severe and very severe aphasias (*p* < .001).

On the ***Reading comprehension subtest***, post hoc analysis revealed that the patients with mild aphasia were significantly better than those with moderate, severe and very severe aphasias (*p* < .001, each). The patients with moderate aphasia were significantly better than those with severe aphasia (*p* = .04) and very severe aphasias (*p* < .001). The patients severe aphasia were significantly better than those with very severe aphasia (*p* = .002).

On the ***Writing subtest***, post hoc analysis revealed that the patients with mild aphasia were significantly better than those with moderate, severe and very severe aphasias (*p* < .001, each). The patients with moderate aphasia were significantly better than those with very severe aphasia (*p* = .01).

Table 10 presents the averages and standard deviations on SAST subtests and on the SAST total score for patients with different types of aphasia.

Table 10 shows that there were significant differences in performance between patients with different types of aphasia on all the subtests of the SAST and on the total SAST scores.

Significant effect of the type of aphasia was detected on the ***total SAST score***. ***Post-hoc analysis*** revealed that the patients with Broca’s aphasia were significantly better than those with Wernicke’s aphasia (*p* = .005), global aphasia (*p* < .001) and unclassified aphasia (*p* < .001). The patients with anomic aphasia were significantly better than those with Broca’s aphasia (*p* = .005), TSA (*p* = .002), Wernicke’s (*p* < .001), global (*p* < .001) and nonspecific (unclassifiable) aphasia (*p* < .001). The patients with TSA were significantly better than those with global aphasia (*p* = .007). The patients with TMA were significantly better than the patients with Wernicke’s (*p* < .001), global (*p* < .001) and nonspecific (unclassifiable) aphasias (*p* < .001). The patients with conduction aphasia were significantly better than the patients with global (*p* < .001) and nonspecific aphasias (*p* = .02). The patients with SMA were significantly better than those with Broca’s aphasia (*p* = .001), TSA (*p* = .001), Wernicke’s aphasia (*p* < .001), global (*p* < .001) and nonspecific aphasia. (*p* < .001).

In addition to the differences in the total score, significant differences between aphasia subgroups on ***each subtest of the SAST*** were also found (Table 10).

Significant effect of the type of was detected on the ***Automatized sequences subtest***. ***Post-hoc analysis*** revealed that the patients with Broca’s aphasia were significantly better than those with global aphasia (*p* < .001) and unclassified aphasia (*p* = .01). The patients with anomic aphasia were significantly better than those with Wernicke’s aphasia (*p* = .01), global aphasia (*p* < .001) and unclassified aphasia (*p* = .01). The patients with Transcortical sensory aphasia (TSA) were significantly better than those with global aphasia (*p* = .01), and nonspecific aphasia (*p* = .04). The patients with Transcortical motor aphasia (TMA) were significantly better than those with global aphasia (*p* = .001) and nonspecific aphasia (*p* = .01). The patients with conduction aphasia were significantly better than the patients with global aphasia (*p* = .04). The patients with SMA were significantly better than those with Wernicke’s aphasia (*p* = .02), global (*p* = .001) and unclassified aphasia (*p* = .02).

On the ***Auditory comprehension subtest***, post hoc analysis revealed that the patients with Broca’s were better than the patients with TSA (*p* = .005), Wernicke’s (*p* = .01) and global aphasia (*p* < .001). The patients with anomic aphasia were better than those with TSA, (*p* = .001), Wernicke’s aphasia (*p* = .002), global aphasia (*p* < .001) and nonspecific aphasia (*p* = .04). The patients with TSA were better than those with global aphasia (*p* = .04). The patients with TMA were better than the patients with TSA (*p* = .002), Wernicke’s aphasia (*p* = .005) and global aphasia (*p* < .001). The patients with conduction aphasia were better than those with TSA (*p* = .003), Wernicke’s aphasia (*p* = .01) and global aphasia (*p* < .001). The patients with SMA were better than those with TSA (*p* < .001), Wernicke’s aphasia *p* = .01), global aphasia (*p* < .001) and with nonspecific aphasia (*p* = .02). The patients with nonspecific aphasia were better than those with global aphasia (*p* = .02).

On the ***Visual naming subtest***, post hoc analysis revealed that the patients with Broca’s aphasia were significantly better than those with global and nonspecific aphasia(*p* = .04). The patients with anomic aphasia were better than those with Broca’s aphasia (*p* = .03), TSA (*p* = .005), Wernicke’s aphasia (*p* < .001), global (*p* < .001) and nonspecific aphasia (*p* < .001). The patients with TMA were significantly better than those with global aphasia (*p* < .001) and nonspecific aphasia (*p* = .02). The patients with SMA were significantly better than the patients with Broca’s aphasia (*p* = .003), and the patients with TSA, Wernicke’s, global and nonspecific aphasias (*p* < .001, each).

On the ***Responsive naming subtest*** post hoc analysis revealed that the patients with Broca’s aphasia were significantly better than the patients with Wernicke’s aphasia (*p* = .005), global (*p* < .001) and nonspecific aphasia (*p* = .05). The patients with anomic aphasia were significantly better than those with Wernicke’s aphasia (*p* = .003), global (*p* < 001) and nonspecific aphasia (*p* = .02). The patients with TMA were significantly better than those with Wernicke’s aphasia (*p* = .004), global (*p* < .001) and nonspecific aphasia (*p* = .03). The patients with SMA were significantly better than patients TSA (*p* = .01), Wernicke’s aphasia (*p* < .001), global (*p* < .001) and nonspecific aphasia (*p* = .001).

On the ***Repetition of words subtest***, post hoc analysis revealed that the patients with Broca’s aphasia were significantly better than the patients with Wernicke’s(*p* = .003), global (*p* < .001) and nonspecific aphasia (*p* = .004). The patients with anomic aphasia were significantly better than those with Wernicke’s aphasia (*p* < .001), global (*p* < .001) and nonspecific aphasia (*p* < .001). The patients with TSA were significantly better than those with Broca’s aphasia (*p* = .04), Wernicke’s aphasia (*p* = .001), global (*p* < .001) and nonspecific aphasia (*p* = .001). The patients with TMA were significantly better than those with Wernicke’s, global and nonspecific aphasias(*p* < .001, each). The patients with conduction aphasia were significantly better than those with global aphasia (*p* = .004).

The patients with SMA were significantly better than those with Wernicke’s, global and nonspecific aphasias (*p* < .001, each).

On the ***Sentence repetition subtest***, post hoc analysis revealed that the patients with Broca’s aphasia were significantly better than the patients with global aphasia (*p* = .004). The patients with anomic aphasia were significantly better than those with Broca’s, conduction, Wernicke’s, global and nonspecific aphasias (*p* < .001, each). The patients with TSA were significantly better than those with Wernicke’s aphasia (*p* = .008), global aphasia (*p* < .001) and nonspecific aphasia (*p* = .002). The patients with TMA were significantly better than those with Broca’s, conduction, Wernicke’s, global and nonspecific aphasias (p < .001, each). The patients with SMA were significantly better than the patients with Broca’s aphasia (*p* = .002), conduction aphasia (*p* = .001), Wernicke’s aphasia (*p* < .001), global (*p* < .001) and nonspecific aphasia (*p* < .001).

On the ***Word reading subtest***, post hoc analysis revealed that the patients with Broca’s aphasia were significantly better than the patients with global (*p* < .001) and nonspecific aphasia (*p* = .007). The patients with anomic aphasia were significantly better than those with Wernicke’s aphasia (*p* = .006), global (*p* < .001) and nonspecific aphasia (*p* < .001). The patients with TMA were significantly better than those with global (*p* < .001) and nonspecific aphasia (*p* = .005). The patients with SMA were significantly better than those with Wernicke’s aphasia (*p* = .003), global (*p* < .001) and nonspecific aphasia (*p* < .001).

On the ***Sentence reading subtest***, post hoc analysis revealed that the patients with Broca’s aphasia were significantly better than the patients with global aphasia (*p* = .02). The patients with anomic aphasia were significantly better than those with Broca’s aphasia (*p* = .05), TSA (*p* = .001), Wernicke’s aphasia (*p* = .004), global (*p* < .001) and nonspecific aphasia (*p* < .001). The patients with SMA were significantly better than those with TSA (*p* = .002), Wernicke’s aphasia (*p* = .006), global (*p* < .001) and nonspecific aphasia (*p* < .001).

On the ***Reading comprehension subtest***, post hoc analysis revealed that the patients with Broca’s aphasia were significantly better than the patients with TSA(*p* = .04), global (*p* < .001) and nonspecific aphasia (*p* = .009). The patients with anomic aphasia were significantly better than those with TSA (*p* = .001), global (*p* < .001) and nonspecific aphasia (*p* < .001). The patients with TMA were significantly better than those with TSA (*p* = .01), global (*p* < .001) and nonspecific aphasia (*p* = .003). The patients with conduction aphasia were significantly better than those with TSA (*p* = .01), Global (*p* = .001) and nonspecific aphasia (*p* = .004). The patients with SMA aphasia were significantly better than those with TSA (*p* < .001), Wernicke’s aphasia (*p* = .006), global (*p* < .001) and nonspecific aphasia (*p* < .001).

On the ***Writing subtest***, post hoc analysis revealed that the patients with anomic aphasia were significantly better than the patients with Broca’s, Wernicke’s, global and nonspecific aphasias (*p* < .001, each). The patients with SMA were significantly better than those with global aphasia (*p* = .03).

In addition to the scored SAST subtests, we compared the participants with different types of aphasia (Broca’s, anomic, etc.) according to the number of words produced during the conversation subtest which was conducted at the start of each assessment session. The results are presented in Table 11.

**Table 11:** Comparison of participants with different types of aphasia according to the total number of words produced in the SAST conversation subtest.

Types of Aphasia	Min	Max	M	SD
1. Broca’s (n = 37)	1	19	10.00	3.84
2. Anomic (n = 7)	19	24	20.29	2.21
3. TSA (n = 7)	8	19	18.14	4.30
4. TMA (n = 13)	6	16	12.08	3.25
5. Conduction (n = 7)	7	17	14.14	3.90
6. SMA (n = 10)	13	24	12.90	3.60
7. Wernicke’s (n = 10)	9	22	16.80	5.01
8. Global (n = 19)	1	6	.32/.95	0.91
9. Nonspecific / Unclassified (n = 10)	0	2	3.70	1.50
F(8, 111)	42.35			
p	< .001			
η^2^	.75			
Significant Scheffe’s post-hoc test	**1**–2, **1**–6, **1**–7, **1**–8, **1**–9; **2**–4, **2**–5, **2**–8, **2**–9; **3**–8, **3**–9; **4**–8, **4**–9; **5**–8, **5**–9; **6**–8, **6**–9; **7**–8, **7**–9			

Note. TMA–Transcortical motor aphasia, SMA–Subcortical motor aphasia, TSA–Transcortical sensory aphasia.

Results of a one-factor analysis of variance showed that patients with different types of aphasias differed with respect to the number of words produced during the conversation SAST subtest ***Post-hoc analysis*** revealed that the patients with Broca’s aphasia produced significantly more words than those with global and nonspecific aphasia. The patients with anomic aphasia produced significantly more words that those with Broca’s, TMA, SMA, global and nonspecific aphasias. The patients with TSA produced significantly more words that those with Broca’s, global and nonspecific aphasias. The patients with TMA and conduction aphasia produced significantly more words that those with global and nonspecific aphasias. The patients with SMA produced significantly more words that those with global and nonspecific aphasias. The patients with Wernicke’s aphasia produced significantly more words that those with Broca’s, global and nonspecific aphasias.

In addition to the types of aphasia, we compared the participants capable of producing fluent spontaneous speech, i.e. the patients with fluent aphasia (anomic, TSA, conduction, and Wernicke’s aphasia) with those with non-fluent aphasia (Broca’s, TMA, SMA, global and nonspecific). The results of the T-test for independent samples shows that the group of patients with fluent aphasia produced significantly more words than the group of patients with non-fluent aphasia (*t* = 8.65, *df* = 118; *p* < .001).

### 3.5. Performance on the SAST in relation to site of lesion

Data presented in Table 12 show that the scores on the SAST subtests and total score depended on the site of the lesion. Patients differed significantly on all subtests other than automatized sequences, word repetition, and writing (*F*s < 2.70, *p*s > .05).

**Table 12:** Differences on SAST subtests and in total score between participants with different site of lesion.

SAST subtests	Automat. sequences	Auditory comprehension	Visual naming	Responsive naming	Repetition of words	Sentence repetition	Word reading	Sentence reading	Reading comprehension	Writing	Total score
Site of lesion	Mean (SD)										
**1** Anterior cortical (n = 20)	1.65 (0.58)	6.35 (1.97)	4.65 (3.29)	1.40 (0.75)	7.20 (3.45)	1.05 (0.69)	6.30 (3.59)	1.05 (0.94)	1.40 (0.75)	0.45 (0.75)	31.95 (12.52)
**2** Posterior cortical (n = 27)	1.30 (0.86)	5.70 (2.55)	3.78 (3.81)	0.93 (0.91)	5.33 (4.17)	0.59 (0.80)	4.33 (4.44)	0.70 (0.91)	0.96 (0.98)	0.52 (0.82)	24.30 (16.48)
**3** Anterior-posterior cortical (n = 66)	1.18 (0.89)	4.88 (2.75)	3.14 (3.66)	0.70 (0.87)	5.20 (4.27)	0.53 (0.72)	3.48 (4.14)	0.44 (0.81)	0.62 (0.84)	0.42 (0.72)	20.58 (16.70)
**4** Subcortical (anterior) (n = 7)	2.00 (-)	7.43 (0.98)	8.71 (2.98)	1.71 (0.75)	8.86 (1.77)	1.43 (0.78)	9.43 (1.51)	1.71 (0.75)	1.86 (0.37)	0.57 (0.97)	43.71 (11.35)
F(3, 116)	2.21	3.24	5.45	5.53	2.75	4.30	6.22	6.45	7.47	0.21	6.29
p	.09	.005	.002	< .001	.04	.007	< .001	< .001	< .001	.86	< .001
η^2^	.05	.77	.13	.12	.07	.10	.14	.14	.16	.01	.15
Significant differences between groups (Scheffe’s post hoc)	-	3–4	2–4; 3–4	1–3; 2–4; 3–4	-	2–4; 3–4	2–4; 3–4	2–4; 3–4	1–3; 3–4	-	2–4; 3–4

Significant effect of the site of lesion variable was detected on the *Auditory comprehension subtest* (*F*(3, 116) = 3.24, *p* = .005). Post-hoc analysis revealed that the patients with subcortical lesions were significantly better than those with anterior-posterior cortical lesions (*p* = .04).

There was also a significant difference between patients with different lesion sites on the *Visual naming subtest*, with post hoc analysis showing that patients with subcortical lesions performed significantly better than those with posterior (*p =* .01) and anterior-posterior cortical lesions (*p =* .003).

On the *Responsive naming subtest*, the post hoc analysis revealed that the patients with anterior cortical lesions were better than those with anterior-posterior cortical lesions (*p =* .02) and the patients with subcortical lesions were better than those with anterior-posterior cortical lesions (*p =* .03).

On the *Sentence repetition subtest*, the post hoc analysis revealed that the patients with subcortical lesions were better than those with posterior cortical lesions (*p =* .04) and the patients with subcortical lesions performed better than those with anterior-posterior cortical lesions (*p =* .005).

On the *Word reading subtest* post hoc analysis showed that patients with subcortical lesions were significantly better than those with posterior cortical lesions (*p =* .03) and patients with subcortical lesions performed better than the patients with anterior-posterior cortical lesion (*p =* .005).

On the *Sentence reading subtest* post hoc analysis showed that the patients with subcortical lesions were better than those with posterior cortical lesions (*p =* .04 and those with anterior-posterior cortical lesion (*p =* .004).

On the *Reading comprehension subtest* post hoc analysis showed that the patients with anterior cortical lesions performed better from those with anterior-posterior cortical lesions (*p =* .008), and that the patients with subcortical lesions were better than those with anterior-posterior cortical lesions (*p =* .006).

The patients also differed on *Total score on the SAST*. Post-hoc analysis revealed that the patients with subcortical lesions were significantly better than patients with posterior cortical (*p* = .04) and anterior-posterior cortical lesions (*p* = .005).

In sum, the site of lesion emerged as a significant predictor of SAST performance, explaining approximately 15% of its variance. Subcortical lesions were associated with the best performance scores on the SAST, followed by anterior cortical lesions. In contrast, both posterior cortical and anterior-posterior cortical were linked to the most pronounced difficulties in SAST performance.

### 3.6. The relationship between SAST and BDAE scores

The final analysis focused on examining the relationship between the SAST and the BDAE subtests scores (see Table 13 in S2 File). The results show a significant correlation between the listed subtests on the SAST and the BDAE battery tests with *r*s ranging between .20 and .80 indicating a substantial overlap between these two assessments.

## 4. Discussion

This study aimed to present the findings of a novel screening test battery for Serbian speakers, called SAST. The battery was developed in response to the need for a language-specific tool to optimize the accurate diagnosis of Serbian-speaking individuals with aphasia. The present study outlined the psychometric properties of the SAST and investigated whether the SAST can distinguish between individual with aphasia and neurotypical individuals. Additionally, the study explored SAST’s potential to detect differences in language and communication skills between different types of aphasia and with respect to the different sites of lesion.

The first set of analyses showed that the SAST had acceptable levels of internal consistency. This is based on the fact that satisfactory to high levels of reliability were achieved (α > .75) on all but one subtest (sentence repetition). Furthermore, the test-retest reliability was excellent. All correlations between the first and second assessments for all subtests were above .85, showing high test-retest reliability. This is in line with Fleiss [26] who proposed that a correlation coefficient of >  .75 meant excellent test-retest reliability.

Moderate to strong correlations between the different SAST subtests provide evidence that all SAST subtests measure the same underlying construct, i.e. the degree of severity of aphasia. The first principal component extracted from the 10 subtest scores explained 78.6% of the common variance. Furthermore, each subscale was highly loaded by the principal component, ranging from .79 to .95, suggesting that the SAST is unidimensional, that is, it measures single latent property of aphasic disorder.

There were also predominantly moderate to strong correlations between the subtests of the Serbian adaptation of the BDAЕ (which is often used to assess the presence and severity of aphasia in Serbian) and the SAST. This finding suggests that the SAST has reasonably good convergent validity and can be used instead of the Serbian adaptation of the BDAЕ.

The second part of the analyses focused on comparing the performance of patients with aphasia and neurotypical controls on the SAST. The two groups differed significantly in all the subtests, and the effect size of the difference was large (e.g., Cohens d was 1.51 for overall score, and it ranged between 0.79 and 6.07 for subtest scores). This suggests that SAST can reliably distinguish between people with aphasia and neurotypical controls.

The performance on the SAST was not predicted by the gender, age or education of the participants with aphasia. This implies that the performance of individuals with aphasia on the SAST is independent of these variables suggesting the potential for standardization irrespective of them. On the other hand, we noted enhanced scores as time elapsed since the injury, providing further evidence of the sensitivity of SAST measures.

By comparing the performance of people with aphasia with different brain damage locations, some differences were observed. The participants with lesions in the subcortical areas differed significantly from those with posterior lesions, and from participants with anterior-posterior cortical lesions. Typically, a comprehensive assessment of aphasia is focused on identifying areas with the most profound impairment. Neuroimaging studies have identified correlations between lesion sites and aphasia [27, 28]. Our data suggest the potential sensitivity of the SAST in discriminating subcortical aphasia (anterior capsular/putaminal aphasia) from aphasia caused by a lesion in the posterior cortex, or aphasia caused by lesions in both the anterior and posterior areas of the cortex. This might be explained by the fact that subcortical lesions have less impact on the severity of language disorders (average values of BDAE scores were the highest for subcortical aphasia, along with anomic aphasia). However, this should be examined further in future studies.

Considering its clinical relevance, SAST can differentiate types of aphasia from each other according to the total score or scores on some subtests. For example, based on the total score SAST can differentiate Broca’s aphasia from anomic, Wernicke’s, and global aphasia, and based on the Sentence repetition subtest, it can differentiate Broca’s aphasia from TMA. Based on the total number of words produced during conversations, the SAST can differentiate fluent from non-fluent aphasia. Regarding its applicability in different phases of aphasia, SAST proved to be useful from the early post-acute stages well into the chronic stages of post-stroke aphasia for quick identification of aphasic symptoms. Finally, the fact that there are significant correlations between scores on BDAE subtests and scores on SAST subtests suggests that the SAST is psychometrically sound and valid as an assessment of language.

We believe that administering a long and elaborate BDAE is not needed, when similar information can be obtained from a shorter and quicker assessment. Compared to the BDAE whose short form takes at least 40 minutes to administer, the SAST can take between 10 and 20 minutes. In the best case, the SAST can be up to four times faster than the short form of the BDAE. Thus, the SAST could be used instead of the BDAE in patients with severe forms of aphasia. For milder forms of aphasia, the BDAE could be used after the SAST to confirm and/or complement the findings from the SAST by providing more detailed information about different linguistic skills. However, the SAST is not a detailed language assessment and, therefore, it cannot provide as comprehensive an insight into specific language deficits (phonological, morphological, or semantical) and more specific linguistically-oriented tests for Serbian need to be developed to provide specific linguistic details.

In addition to taking more time than the SAST, the BDAE does not have psychometric characteristics for the Serbian population, and it has not been formally adapted for the Serbian socio-cultural context. It remains a simple translation, not a real adaptation.

## 5. Conclusions

This study aimed to establish the validity and reliability of a new screening test, the SAST, for native speakers of Serbian with aphasia. Overall, our findings demonstrate that the SAST reliably identifies acquired aphasia in Serbian speakers, differentiates levels of severity, and can highlight differences in the profile of aphasic disorders, thereby confirming its clinical relevance. It is a short and simple measure which allows clinicians to detect the presence of aphasia and language difficulties associated with aphasia.

The test is also, potentially, able to distinguish between language difficulties resulting from different lesion locations. We believe that our test is a promising new assessment tool, which can enable clinicians to quickly identify the presence of aphasic syndromes in people after a brain injury This should provide the opportunity for remediation and rehabilitation to be planned early to optimise language recovery outcomes.

## Supporting information

S1 File SAST (in Serbian and an English translation). (DOCX)

S2 File Table 13: Relationship between the SAST and the BDAE subtests scores. (DOCX)

S3 File Raw data and variables description. (XLSX)
