# Supplementary material for: Evaluation of reliability and validity of the Serbian Aphasia Screening Test
Source: PLoS One. 2024 May 31;19(5):e0304565. doi: 10.1371/journal.pone.0304565 (PMC11142518; doi:10.1371/journal.pone.0304565)
Supplement: S1 File — (DOCX) [file pone.0304565.s001.docx]

**СКРИНИНГ ТЕСТ ЗА АФАЗИЈЕ (ВУКОВИЋ, М., 2010)**

Пацијент: ___________________________ Старост: _________________________

Бр. година школовања: ________ Занимање: ________________________ Доминантност руке : __________________________________________________

Употреба језика: Први језик _____________ Други језик ______________

Тип неуропатологије (цереброваскуларни инсулт, траума мозга, тумор, друго): __________________________________________________________

Место лезије: ____________________Датум настанка: __________________

*Конверзација*:

Реците ми Ваше име и презиме.

Где живите?

Шта сте по занимању?

Реците ми шта Вам се догодило, због чега сте дошли код нас?

Остварени поени Могући поени

*Вербални аутоматизми*:

1. Бројте до 21. __ 1
2. Набројте дане у недељи. __ 1

*Разумевање:*

1. Покажите нос. __ 1

2. Покажите уво. __ 1

3. Затворите очи. __ 1 4. Левом руком додирните десно уво^1^. __ 2 5. Стегните песницу, подигните руку и притом жмурите. __ 3

*Именовање предмета^2^* : сат (673), кључ (33), чешаљ (9), оловка (34), прстен (32), дугме (40), спајалица (0), упаљач (1), новац (227), телефон (38) __ 10

Именовање изазвано питањима:

1. Чиме сечемо хлеб? ______________ __ 1 2. Чиме перемо зубе? ______________ __ 1

__________________________________________________________________________________

^1^ У случају леве хемипарезе, инструкција гласи: Десном руком додирните лево уво.

^2^ Фреквенције речи су дате у заградама.

**Скрининг тест за афазије**

**_________________________________________________________________________________**

*Понављање:*

а) речи: со, пас, сто, код, штап, камин, лимун, књига, брод, кључ __ 10 б) реченице: 1. Данас је лепо време. __ 1 2. Попео се степеницама на први спрат и затворио лифт. __ 1 *Читање:*

а. Речи: сат, клупа, браон, десет, мајка, тигар, слаб, пије, тих, спава __ 10 б. Реченице:

1. Ветар се стишао. __ 1

2. Голуб је слетео на прозор његове собе. __ 2

*Разумевање прочитаног:*

1. Отворите уста. __ 1 2. Подигните леву руку.^3^ __ 1

Писање:

1. Напишите своје име и презиме: _____________________ __ 1 2. Напишите реченицу коју сами смислите: __________________________________________________ ` __ 1

Укупни скор Могући скор:

_______________ 50 поена

Датум испитивања: _______________________________

Тип афазије: _____________________________________

Испитивач: ______________________________________

__________________________________________________________________________________

^3^ У случају леве хемипарезе, инструкција гласи: Подигните десну руку

**SERBIAN APHASIA SCREENING TEST - SAST**

**(VUKOVIĆ, M., 2010)**

Patient: ___________________________________ Age: _________________________

Years of education: ______________ Occupation: _________________________

Handedness history: ____________________________________________________

Languages: First Language _____________ Second Language ____________

Neurological disease / impairment (stroke, brain trauma, tumour, other): ____________________________________________________________________

Site of lesion: _________________________ Date of onset: __________________

*Conversation*:

What is your full name?

Where do you live?

What do you do for living?

Could you tell me what happened to you, and why did you come to us?

Points scored Maximum

*Automatized sequences:*

1. Count to 21. __ 1

2. Name the days of the week. __ 1

*Comprehension of* a*uditory commands*

1. Point to your nose. __ 1 2. Point to your ear. __ 1 3. Close your eyes. __ 1 4. Touch your right ear with your left hand.^1^ __ 2 5. Make a fist, raise your arm and close your eyes. __ 3

*Visual naming*^2^ :

Watch (673), key (33), comb (9), pencil (34), ring (32), button (40), paperclip (0), lighter (1), banknote (227), telephone (38) __ 10

*Responsive naming*:

1. What do we cut bread with? __________________ __ 1 2. What do we brush our teeth with? ____________ __ 1

_________________________________________________________________________________

^1^ In the case of left hemiparesis: “Touch your left ear with your right hand.”

^2^ Frequency of words is given in parentheses.

**Serbian Aphasia Screening Test – SAST**

**__________________________________________________________________________________**

Points scored Maximum

*Repetition:*

1. of words: salt, dog, table, at, stick, fireplace, lemon, book, key, ship __ 10 b) of sentences:

1. The weather is nice today. __ 1 2. He climbed the stairs to the first floor and closed the lift. __ 1

*Reading:*

a. of words: watch, bench, brown, ten, mother, tiger, weak, drinks (verb), quiet, sleeps (verb) __ 10 b. of sentences:

1. The wind calmed down.

2. A pigeon landed on his bedroom window. __ 2

*Understanding of written language:*

1. Open your mouth. __ 1

2. Lift your left arm^3^ __ 1

*Writing:*

1. Write down your full name: ___________________________ __ 1

2. Write down any sentences you can think of: ____________________________________________________ __ 1

Total score Max score: ______________ 50 pts

Date of testing: __________

Type of aphasia: __________

Examiner: _______________

__________________________________________________________________________________

^3^In the case of left hemiparesis: “Lift your right arm.
