# Supplementary material for: Evaluation of reliability and validity of the Serbian Aphasia Screening Test
Source: PLoS One. 2024 May 31;19(5):e0304565. doi: 10.1371/journal.pone.0304565 (PMC11142518; doi:10.1371/journal.pone.0304565)
Supplement: S2 File — (DOCX) [file pone.0304565.s002.docx]

**Table 13.** *Correlations between the SAST and the Serbian translation of the BDAE*

| **BDAE**  **SAST** | Total number of words  (first 7 – Part Conversation) | Automatized sequences | Auditory comprehen-sion of commands | Visual confronta-tion naming | Responsive naming | Repetition of words | Sentence repetition (high probability) | Word reading | Oral sentence reading | Reading sentences and paragraphs | Sentences written to dictation |
| --- | --- | --- | --- | --- | --- | --- | --- | --- | --- | --- | --- |
| Total number of words | .75*** | .72*** | .46*** | .68*** | .51*** | 57*** | 44*** | .55*** | .60*** | .48*** | .38*** |
| Automatized sequences | .26** | .41*** | .64*** | .46*** | .48*** | .36*** | .30** | .42*** | .33** | .30** | .21* |
| Auditory comprehension | .24* | .63*** | .66*** | .52*** | .46*** | .28* | .29** | .44*** | .42*** | .41*** | .20* |
| Visual confron-tation naming | .41*** | .56*** | .64*** | .81*** | .68*** | .60*** | .51*** | .70*** | .69*** | .58*** | .34*** |
| Responsive naming | .23* | .57*** | .60*** | .65*** | .64*** | .48*** | .39*** | .51*** | .47*** | .36*** | .19* |
| Repetition of words | .44*** | .57*** | .59*** | .70*** | .65*** | .73*** | .63*** | .61*** | .56*** | .33** | .26* |
| Repeating of sentences | .46*** | .59*** | .62*** | .68*** | .66*** | .75*** | .79*** | .61*** | .58*** | .52*** | .26* |
| Word reading | .50*** | .62*** | .61*** | .73*** | .66*** | .61*** | .54*** | .74*** | .73*** | .51*** | .35** |
| Oral sentence reading | .34*** | .50*** | .65*** | .68*** | .67*** | .44*** | .39*** | .61*** | .80*** | .52*** | .32*** |
| Reading comprehension | .40*** | .45*** | .47*** | .63*** | .55*** | .37*** | .37*** | .67*** | .67*** | .50** | .30** |
| Writing | .33** | .47*** | .39*** | .51*** | .47*** | .31*** | 37*** | 51*** | .51*** | .51*** | .48*** |

*Note*. ^*^p <.05, ^**^p< .01, ^***^p< .001
